# Fermentation Quality, In Vitro Digestibility, and Aerobic Stability of Total Mixed Ration Silage in Response to Varying Proportion Alfalfa Silage

**DOI:** 10.3390/ani12081039

**Published:** 2022-04-16

**Authors:** Yixiao Xie, Lei Wang, Wenqi Li, Shengyang Xu, Jinze Bao, Jiajie Deng, Zhe Wu, Zhu Yu

**Affiliations:** 1 College of Grassland Science and Technology, China Agricultural University, Beijing 100193, China; xieyx@gzu.edu.cn (Y.X.); wanglei938210@163.com (L.W.); lwqnd@cau.edu.cn (W.L.); 82101211217@caas.cn (S.X.); sy20183040590@cau.edu.cn (J.B.); wuzhe@cau.edu.cn (Z.W.); 2 College of Animal Science, Guizhou University, Guiyang 550025, China; 3 State Key Laboratory of Animal Nutrition, College of Animal Science and Technology, China Agricultural University, Beijing 100193, China; dengjj@cau.edu.cn

**Keywords:** TMR silage, alfalfa silage, fermentation quality, in vitro digestibility, aerobic stability

## Abstract

**Simple Summary:**

Ensiling a total mixed ration (TMR) allows longer storage times for thoroughly mixed feed. In our previous study, we found that alfalfa silage could directly provide an acidic environment in fresh TMR and had a function comparable to that of lactic acid bacterial inoculants in improving fermentation quality during ensiling. However, the specific percentage of silage addition to the TMR has not been evaluated. In this study, we explored the effects of different proportions of alfalfa silage on the fermentation quality, in vitro digestibility, and aerobic stability of TMR silage and discussed the results in terms of the chemical composition of the feed. We found that the gradual development of an acidic environment during the ensiling process, which can be achieved with 40% alfalfa silage addition, can improve aerobic stability. However, the addition of 60% or 20% alfalfa silage might cause lower aerobic stability or clostridial spoilage in TMR silage. Our findings provide a more in-depth understanding of the effects of silage components on TMR silage and may guide farmers to apply a proper silage composition in rations to avoid the loss of feed value during storage or feeding stages.

**Abstract:**

This study aimed to evaluate the effects of different proportions of alfalfa silage on the fermentation quality, in vitro digestibility, and aerobic stability of total mixed ration (TMR) silage. Three TMRs were prepared with different silage contents on a fresh matter basis: (1) 60% alfalfa silage (AS60), (2) 40% alfalfa silage (AS40), and (3) 20% alfalfa silage (AS20). The lactic acid in AS60 did not increase after 30 days of ensiling (*p* > 0.05). Butyric acid was detected in the AS20 group after 14 days of ensiling. The AS60 group showed significantly higher in vitro dry matter digestibility than the AS20 group (*p* < 0.05). The aerobic stability of TMR silage gradually increased with a decreasing percentage of alfalfa silage (*p* < 0.05). Unlike AS60, which directly gained an acidic environment from the alfalfa silage, AS40 developed a stable acidic environment during ensiling and further improved aerobic stability. However, when the percentage of alfalfa silage was reduced to 20%, a risk of clostridial spoilage occurred in the TMR silage. Therefore, the addition of 40% alfalfa silage to TMR is optimal and could achieve both good fermentation quality and considerable resistance to aerobic deterioration in TMR silage.

## 1. Introduction

Total mixed ration (TMR) is a feed that mixes forages, byproducts, concentrates, minerals, vitamins, and other additives in a way that provides enough nutrients to meet the needs of ruminants. TMR silage has been studied since the 1960s in the United States [1], and it has been a focus of research in many countries in recent years [2]. Ensiling TMR for ruminants allows longer storage time and higher aerobic stability for thoroughly mixed feed. Anaerobic fermentation can potentially improve the palatability of unpalatable byproducts if their odors and flavors are altered. The commercialization of TMR silage reduces the requirements for labor and machinery when TMR silage bales are purchased by small-scale farms [2].

Alfalfa (*Medicago sativa* L.) is an important forage crop species with high yields in Ningxia and worldwide, and it can provide a great balance of energy, protein, and minerals for livestock production [3]. Alfalfa silage is highly nutritive for ruminants because of its relatively low fiber content, high protein content, and high digestibility [3,4]. In Ningxia, alfalfa frequently cannot be harvested in the proper period (the budding stage to the early bloom stage) due to the local rainy season, resulting in a decline in the quality of alfalfa silage [5]. A portion of protein feed can sometimes be replaced with relatively inexpensive alfalfa silage of poor quality in TMR production. Alfalfa silage is favorable for ruminal fermentation [4,6], and well-fermented forage silage in TMR can effectively mask and dilute the flavors of unpalatable ingredients [7]. By adjusting the proportion of alfalfa silage, the protein content of TMR silage can be controlled to meet the nutritional needs of cattle at different growth stages.

However, similar to other silage processes, TMR silage fermentation does not always proceed smoothly. Improper formulations may cause undesirable fermentation during ensiling. Adequate lactic acid fermentation of TMR silage depends on the dry matter (DM) content, soluble carbohydrate content, buffering capacity, and microbial community, especially the population of lactic acid bacteria (LAB) in the TMR [2]. Furthermore, TMR silage exposed to air may also undergo aerobic deterioration if not fed soon after unpacking. Small-scale family farms may take several days to consume large TMR silage bales. Adjusting the proportions of specific ingredients in the formula can also greatly affect the aerobic stability of TMR silage [8]. In our previous study, we found that well-fermented alfalfa silage containing adequate lactic acid (or LAB) had a comparable function to LAB inoculants in the improvement of ensiling [5]. However, the specific percentage of alfalfa silage addition to the TMR has not been evaluated. Thus, the effects of different proportions of alfalfa silage on the fermentation quality, in vitro digestibility, and aerobic stability of TMR silage were explored in this study.

## 2. Materials and Methods

### 2.1. Total Mixed Ration (TMR) Silage Preparation

The chemical composition and fermentation characteristics of the ingredients of the TMR are given in Table 1. First-cut alfalfa (*Medicago sativa* L, “Longdong”) was harvested at the full-bloom stage in Guyuan, Ningxia, China (106°17′ E, 36°28′ N, elevation 1529 m) in mid-June. The wilted alfalfa was chopped to 2–5 cm, baled by a round baler (Comprima, Krone, Germany), and inoculated with *Lactobacillus plantarum* (LP). The application rate of LP was 10^6^ colony-forming units (cfu)·g^−1^ of fresh matter (FM). The water-soluble carbohydrate (WSC) content in the alfalfa was 79.68 g·kg^−1^ DM. The buffering capacity of the fresh alfalfa was 310 mEq·kg^−1^ DM. Baled alfalfa silage was unpacked after 55 days of ensiling. Corn cobs, corn grain, and mixed concentrate were collected from a medium-scale beef cattle farm in Guyuan, Ningxia, China. The mixed concentrate was produced by Botai Company (Guyuan, China) and composed of rapeseed meal, soybean meal, cotton meal, corn gluten meal, corn peel, dried distiller’s grains, and vitamin–mineral mix.

The formulations and chemical compositions of the TMR before ensiling are given in Table 2. Approximately 750 g of TMR mixture (FM) was thoroughly mixed and then tightly packed into 1 L plastic silos. The three TMR formulas were designed according to *Nutrient Requirements of Beef Cattle* [9] and contained 60% (AS60), 40% (AS40), and 20% (AS20) alfalfa silage. The formulations of AS60, AS40, and AS20 groups were designed with total digestible nutrients of 694.9, 699.9, and 704.9 g·kg^−1^ DM, respectively, which could meet the nutritional needs of 250 kg, 300 kg, and 400 kg fattening cattle, providing a daily gain of 1 kg each. In total, 15 silos containing each formula were prepared, with triplicate silos opened on day 7 and day 14 to determine the fermentation quality of the TMR silage, while the other nine silos were opened on day 30 to determine both the fermentation quality and aerobic stability of the TMR silage. The silos were stored in a room at 22–28 °C. In addition, at the time of ensiling, triplicate silos containing well-mixed fresh TMR of each formula were used as 0 day TMR silages for analysis.

### 2.2. Chemical and Microbiological Analyses

At the time of opening, the TMR silages were removed and blended thoroughly. Approximately 200 g TMR silage samples were analyzed for DM content by oven-drying at 65 °C for 48 h [10]. The dried samples were ground with a mill and passed through a 1 mm screen for further chemical analyses. The total nitrogen (TN) was determined according to the Kjeldahl procedure [11], and the crude protein (CP) content was calculated as TN × 6.25. The content of WSCs was determined by the anthrone–sulfuric acid method [12]. Neutral detergent fiber (aNDF) and acid detergent fiber (ADF) were determined according to Van Soest et al. [13]. Sodium sulfite and α-amylase were applied for aNDF determination, and both the aNDF and ADF contents reported included residual ash.

A subsample of 20 g from each silo was mixed 1:9 (*w*/*v*) with distilled water and then homogenized in a blender jar, followed by filtration through four layers of cheesecloth and filter paper. The pH of this filtrate was measured by an electrode (PHS-3C, INESA Scientific Instrument, Shanghai, China). After centrifugation (10,000× *g*, 4 °C, 10 min), the filtrate supernatant was used to determine the ammonia nitrogen (NH_3_-N) and organic acid (lactic acid, acetic acid, propionic acid, and butyric acid) contents. The NH_3_-N content was determined by the sodium hypochlorite and phenol method [14]. Organic acid contents were determined by high-performance liquid chromatography as described by Tian et al. [15]. The V-score, which is used to evaluate the silage quality, was calculated according to the volatile fatty acid and NH_3_-N contents [16]. Microorganism (LAB, yeasts, molds, and coliform bacteria) numbers were determined by the plate count method as described by Wang et al. [17].

### 2.3. In Vitro Incubation and Degradability Measurement

Rumen fluid was collected from four fistulated Holstein dairy cows fed 11 kg (FM) of whole-plant corn silage, 3.5 kg of wheat straw, 2.8 kg of oat hay, 1.3 kg of corn peel, 1 kg of soybean meal, 1 kg of rapeseed meal, 0.5 kg of dried distiller’s grains with solubles, 0.05 kg of urea, and 0.2 kg of 5% premix. The rumen fluid was filtered and kept at 39 °C. Filtered rumen fluid was mixed with a buffer solution at a ratio of 1:4 as described by Xie et al. [5]. In vitro gas production and in vitro digestibility analyses were also carried out as described previously [5]. Briefly, gas production was recorded by Ankom RFS bottles using the pressure transducer technique (Ankom Technologies, Macedon, NY, USA). Approximately 1 g of ground 30 day TMR silage sample was mixed with 125 mL of fluid–buffer mixture. The mixtures were incubated at 39 °C, and gas production was recorded every hour for 48 h. Three RFS bottles containing only inoculant were incubated and recorded as blanks, and all in vitro determinations were carried out in two separate runs. Cumulative gas production data were fitted to the model modified from the Gompertz growth equation [18].

V(*t*) = V(∞) exp [–exp (*ke* (λ − *t*)/V(∞) + 1)], (1)

where V*(t)* is the cumulative gas production (mL), V(∞) is the maximal cumulative gas production (mL), *k* is the maximum gas production rate (mL·h^−1^), λ is the lag time (h), *t* is the time elapsed (h), and *e* is an exponential of one (2.718).

An Ankom Daisy^II^ incubator (Ankom Technologies, Macedon, NY, USA) was used to determine the in vitro digestibility of the 30 day TMR silages. Approximately 0.5 g of ground sample was placed into an artificial filter bag (F57, Ankom Technologies, Macedon, NY, USA) and incubated in the same fluid–buffer mixtures under CO_2_ for 48 h. After incubation, the artificial filter bags were collected, and the weight reductions of the bags were recorded to calculate the in vitro dry matter digestibility (DMD). The aNDF contents of the residue were also determined to calculate the in vitro neutral detergent fiber digestibility (NDFD).

### 2.4. Aerobic Stability Test

After 30 days of ensiling, the remaining nine silos corresponding to each formula were opened at the same time to ensure sufficient TMR silages for sampling in the aerobic exposure test. Sets of three silos were mixed and taken as a 30 day TMR silage sample (with the same procedure used for the 0 day aerobic exposure samples) for chemical analyses and in vitro incubation. The remaining silages were loosely packed into four new 1 L plastic silos and marked with different sampling times (days 1, 3, 5, and 7). A total of 36 silos (3 formulas × 3 replicates × 4 sampling days) were prepared for the aerobic stability test. All silos were covered with four layers of cheesecloth and stored at an ambient temperature of approximately 25 °C. The temperature of the exposed TMR silages was measured and recorded every half-hour for 7 days by a multichannel data logger (MDL-1048A, Tianhe, Shanghai, China). Aerobic stability was denoted as the time (h) interval before the silage temperature increased by 2 °C above the ambient temperature. Silages subjected to aerobic exposure were sampled at 0, 1, 3, 5, and 7 days to determine the pH, organic acid, NH_3_-N, DM, CP, and WSC contents, and microbial counts.

### 2.5. Statistical Analyses

Microbial data were log_10_-transformed and presented on a fresh matter basis. The data were subjected to one-way or two-way analysis of variance with fixed effects of ensiling time (or exposure time) and varying proportions of alfalfa silage using SPSS version 19.0 for Windows (SPSS Inc., Chicago, IL, USA). Duncan’s multiple range test was conducted to determine the differences among means between different formulas and ensiling days (or exposure days). Significance was declared at *p* < 0.05. The gas production kinetic parameters V(∞) and *k* were estimated by an iterative least squares method using nonlinear regression in SPSS.

## 3. Results

### 3.1. Fermentation Quality of TMR Silages

The fermentative characteristic changes in the TMR silages during 30 days of ensiling are shown in Table 3. The interaction between treatments and ensiling days significantly affected (*p* < 0.05) the pH, lactic acid, acetic acid, butyric acid, and NH_3_-N levels, and V-score. The effects of different treatments on all the fermentative characteristics of the TMR silages were evident (*p* < 0.05). The ensiling period also had a significant effect (*p* < 0.05) on all fermentative characteristics except the acetic acid content.

After 14 days of ensiling, the pH of the AS40 group was significantly lower than that of the other two groups (*p* < 0.05). Compared to day 7, the pH value of the AS20 group was increased at day 14 (*p* < 0.05). There was no significant change in the pH or lactic acid content in the AS60 group at day 30 of fermentation compared to that at day 0 (*p* > 0.05). In contrast, both the AS20 and AS40 groups showed higher lactic acid contents after 30 days of ensiling (*p* < 0.05). The acetic acid and propionic acid contents of the TMR silages significantly increased after 30 and 14 days of ensiling, respectively (*p* < 0.05). Butyric acid was not detected in either the AS60 or AS40 groups but was detected in the AS20 group after 14 days of ensiling. Moreover, the AS20 group also showed a significantly higher NH_3_-N content than the AS40 and AS60 groups during days 14–30 (*p* < 0.05). The V-score of the AS20 group significantly decreased during days 14–30 and was lower than that of the other two groups (*p* < 0.05). In addition, the AS20 silages showed lower WSC contents (*p* < 0.05) and much higher DM losses (*p* < 0.05) than the other silages did after 30 days of ensiling (Table 4).

### 3.2. In Vitro Degradability of TMR Silages

**Figure 1:**
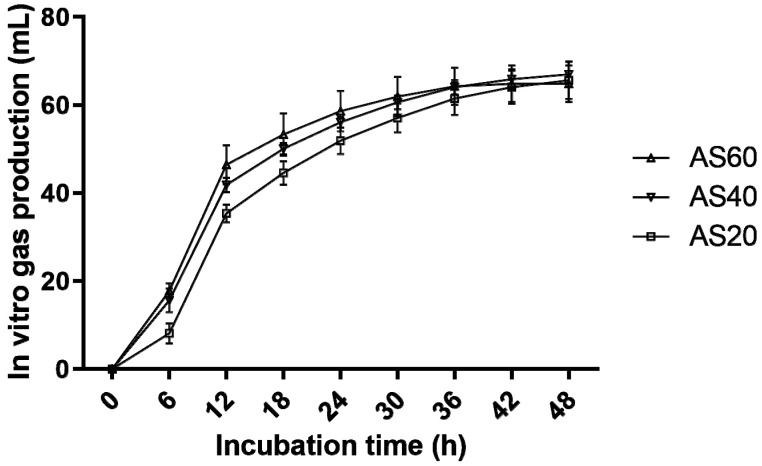
Gas production (mL) profiles from in vitro fermentation of the TMR silage for 48 h (bars indicate the standard errors of the means). AS60, TMR containing 60% alfalfa silage (DM); AS40, TMR containing 40% alfalfa silage (DM); AS20, TMR containing 20% alfalfa silage (DM).

The in vitro gas production profiles and parameters of the 30 day TMR silages are presented in Figure 1 and Table 4, respectively. The curves of gas production tended to plateau by 48 h; thus, the gas production kinetic parameters were also calculated. During the first 18 h of the in vitro gas production test, the gas production rate of the AS20 silages tended to be slower than those of the AS40 and AS60 silages. However, the differences in the maximum gas production rate and 24 h and 48 h cumulative gas production among the three formulas were not significant (*p* > 0.05). The AS60 silages showed higher DMD than the AS20 silages (*p* < 0.05), but there were no differences in NDFD among the three formulas (*p* > 0.05).

### 3.3. Aerobic Stability of TMR Silages

The fermentative characteristics and chemical composition changes of the TMR silages during the aerobic exposure period are shown in Table 5. Significant interactions between the different treatments and days of aerobic exposure were observed for the pH, lactic acid, acetic acid, DM, and NH_3_-N levels, and V-score (*p* < 0.05). The treatments showed significant effects on all fermentative characteristics and chemical compositions of the TMR silages (*p* < 0.05). The days of aerobic exposure significantly affected the pH, lactic acid, NH_3_-N, DM, and WSC levels, and V-score (*p* < 0.05).

Only the pH of AS20 silages remained stable during the whole aerobic exposure test (*p* > 0.05). However, the pH of the AS60 and AS40 silages significantly increased after 5 days of aerobic exposure (*p* < 0.05), reaching 7.29 and 6.86, respectively, on the seventh day. Correspondingly, the lactic acid contents of the AS60 and AS40 silages significantly decreased after 7 days of aerobic exposure (*p* < 0.05). The propionic acid content of the AS60 silages was significantly higher than that of the AS40 and AS20 silages (*p* < 0.05). No butyric acid was detected in either the AS60 or AS40 silages. The CP content of the AS40 group decreased and the NH_3_-N contents of the AS60 and AS40 significantly increased after 7 days of aerobic exposure (*p* < 0.05); thus, the V-scores of the two groups significantly decreased on the seventh day (*p* < 0.05).

**Figure 2:**
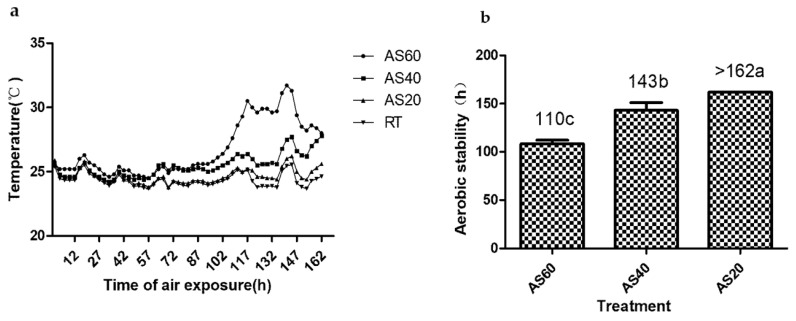
Dynamic changes in temperatures (**a**) and hours of aerobic stability (**b**) of the TMR silages during air exposure (bars indicate the standard errors of the means). Values with different letters show significant differences among the treatments (*p* < 0.05). AS60, TMR containing 60% alfalfa silage (DM); AS40, TMR containing 40% alfalfa silage (DM); AS20, TMR containing 20% alfalfa silage (DM); RT, room temperature.

As presented in Figure 2a, the AS60 silages showed an earlier and greater temperature rise than the other two groups. The TMR silages showed aerobic stabilities ranging from 110 to >162 h (Figure 2b). The aerobic stabilities of the three formulas differed significantly from each other (*p* < 0.05). Table 6 shows the dynamic changes in viable microbial counts during 7 days of aerobic exposure. The AS40 silages showed higher LAB counts on the seventh day of the aerobic exposure test than the other silages (*p* < 0.05). The yeast counts of both the AS60 and AS40 silages reached 10^5^ cfu·g^−1^ (FM) after 3 days of aerobic exposure. In contrast, the yeast counts of the AS20 group remained under 10^4^ cfu·g^−1^ (FM) until the end of the aerobic exposure test. The AS20 silages showed lower mold, yeast, and coliform bacterial counts than the other two formulas on the seventh exposure day (*p* < 0.05). Molds were detected in the AS60 and AS40 groups on the third and seventh days, respectively, but no molds were detected in the AS20 silages.

## 4. Discussion

### 4.1. Effects of Varying Proportions of Alfalfa Silage on the Fermentation Quality of TMR Silages

The high percentage of alfalfa silage may have directly produced an acidic environment in the AS60 silages. Therefore, the lactic acid content and pH in the AS60 group did not change significantly during ensiling, which is consistent with our previous findings [5]. In contrast, the lactic acid contents in the AS40 and AS20 groups gradually increased during ensiling due to the lower original silage contents. The lactic acid contents of TMR silage in the AS40 and AS60 groups, which mainly originated from alfalfa silage, tended to decrease on the seventh day of ensiling. This result may be due to residual oxygen at the beginning of fermentation promoting the growth of aerobic microorganisms (such as molds, yeasts, and aerobic bacteria), which can use lactic acid and other volatile fatty acids for metabolism [19]. Moreover, previous studies showed that re-ensiled silage may have a lower NH_3_-N content than normal silage [5,19]. A similar result was found in the present study, in which the NH_3_-N content tended to decrease after 7 days of ensiling. All TMR silages in the present study showed higher V-scores on the seventh day due to lower NH_3_-N, acetic acid, and propionic acid contents.

Butyric acid was detected in the AS20 silages after 14 days of ensiling. In the experiment conducted by Wang et al. [20], butyric acid was also detected in alfalfa silage on the 14th day. This may be due to the occurrence of clostridial fermentation [21]. Silage clostridia have both saccharolytic and proteolytic properties [22]. Clostridia not only use WSCs, lactic acid, and acetic acid as substrates to produce butyric acid but also decarboxylate free amino acids to produce amines and NH_3_ [22]. Clostridia generally thrive in low-sugar silages with high moisture contents (>70%), pH values (>4.6), temperatures (>30 °C), and buffering capacities [23]. The contents of WSCs and DM are considered the key factors that decrease the pH and inhibit clostridia in silage [22]. Researchers have found that the optimal DM content of TMR is 45–60% [7]. Therefore, the TMR silages in the present study exhibited high DM contents, although all had low sugar contents (12–15 g·kg^−1^ DM). A higher DM content could reduce the availability of inorganic ions to form a buffer system with the weak organic acids produced in the silage and facilitate the fermentation of silage with a low carbohydrate content [24]. Hao et al. [25] found little difference in fermentation quality between TMR silages with DM contents of 51.7% FM and 56.8% FM when the WSC content of the feedstock reached 62–65 g·kg^−1^ DM, and no butyric acid was detected in any samples. Therefore, the production of butyric acid in the AS20 group might be due to the lack of sufficient WSCs for fermentation under slightly higher dry matter conditions (53.7% vs. 50.1% FM). On the other hand, the differences in the acidic environment and LAB population caused by different alfalfa silage contents might also have an important impact on the fermentation quality. Compared to the AS20 silages, the AS40 silages predominately showed lactic acid fermentation, although the initial WSC contents of the TMR were also low, which was likely due to the higher alfalfa silage content exerting a stronger inoculant-like effect.

The pH of the AS20 group did not continue to decrease after 14 days of ensiling, while the NH_3_-N content greatly increased probably due to the proteolytic properties of clostridia, creating a high buffering capacity in these TMR silages [26]. The pH decrease due to lactic acid fermentation also depends on the buffering capacity of the crop [27]. A higher buffering capacity will prevent a pH decrease in silage, even if the silage has a high lactic acid level. Furthermore, the conversion of lactic acid to H_2_, CO_2_, and butyric acid by clostridia also leads to an increase in the silage pH [21,22]. A fermentation environment with a high pH will further promote the growth of clostridia. Clostridial spoilage in the AS20 group after 14 days of fermentation caused increases in NH_3_-N and butyric acid contents, which led to a rapid decrease in the V-score. The V-scores of the AS40 and AS60 groups without clostridial fermentation remained stable in the later stages of anaerobic fermentation. Therefore, it may be necessary to apply additional LAB inoculants or fermentable substrates in the AS20 group to promote lactic acid fermentation and inhibit the occurrence of clostridial spoilage.

### 4.2. Effects of Varying Proportions of Alfalfa Silage on the In Vitro Degradability of TMR Silages

Digestibility can be used to evaluate the nutritional value and intake of animal feed [8]. In addition, in vitro gas production is an indicator of feed digestibility [28], which can be used to predict the metabolizable energy of TMR silage [8]. Du et al. [29] investigated the relationship between the chemical composition of forage and the ruminal degradation of nutrients, and the results showed that DMD and NDFD were positively correlated with the CP content and negatively correlated with the NDF and ADF contents. Several studies have investigated the relationship between the CP level and gas production, and inconsistent and conflicting results have been obtained [30,31,32,33]. However, the literature results on the relationship between NDF and gas production are relatively uniform and indicate a negative correlation [32]. Although the improvements in the cumulative gas production and gas production rate in the AS60 silages were not significant, the AS60 silages with higher alfalfa silage contents showed significantly higher DMD than other TMR silages, which may be due to the higher CP contents and lower NDF contents. Therefore, in the present study, an increased percentage of alfalfa silage in TMR had a positive effect on the digestibility of the feed.

### 4.3. Effects of Varying Proportions of Alfalfa Silage on the Aerobic Stability of TMR Silages

Some small-scale family farms may take more than 5 days to consume a large TMR silage bale (more than 800 kg, FM). Therefore, it is necessary to ensure sufficient aerobic stability of TMR silages. Aerobic deterioration of silage involves the loss of sugars, the generation of heat, and the evolution of NH_3_ and CO_2_ [34,35]. Fungi and yeasts in particular usually play large roles in aerobic deterioration in silage [36]. However, the yeast counts in the AS20 silages were always below 10^4^ cfu·g^−1^, and no molds were detected during the 7 days of aerobic exposure. Moreover, the temperature of the AS20 silages did not increase until the end of the test. This might be due to the presence of large amounts of butyric acid in the AS20 silages, which is more antimycotic than acetic acid and inhibited the growth of aerobic microorganisms [37]. Danner et al. [38] found that the aerobic stability of silage was significantly improved when it contained small amounts of butyric acid (>5 g·kg^−1^ DM). Therefore, less lactic acid was metabolized by undesired microorganisms in the AS20 silages, which maintained stable pH, CP, and WSC levels on the seventh day of exposure. Although the antifungal properties of butyric acid could improve the aerobic stability of TMR silage, its presence could also cause a reduction in DM intake [39] and increase the probability of ketosis within the herd [23]. Due to the presence of butyric acid, the V-scores of the AS20 silages were always very low, even though they did not continue to decrease during aerobic exposure. In contrast, the AS40 and AS60 groups had significant decreases in V-scores only on the seventh day of the exposure test due to increased NH_3_-N contents caused by proteolysis.

In the two treatment groups with higher alfalfa silage contents, the increased proportions of alfalfa silage caused increases in the acetic acid and propionic acid contents, and this result is similar to that of Wang et al. [20,40]. The growth of clostridia in the AS20 group before aerobic exposure may have caused an increase in the acetic acid content through redox reactions of amino acids or the breakdown of lactic acid [22], resulting in a slightly higher acetic acid content than that of the AS40 group. Although acetic and propionic acid contents generally contribute to better aerobic stability, their levels were low in all treatment groups in the present study. In a study by Danner et al. [38], the improvement of aerobic stability by acetic acid was very limited when the acetic acid content was below 15 g·kg^−1^ DM. Although the AS60 group had a higher propionic acid content, its aerobic stability was not better than that of the AS40 group. Therefore, the difference in aerobic stability between the AS60 and AS40 groups was likely not caused by differences in the VFAs of the silage. After exposure to air, lactic acid (produced during ensiling or provided by alfalfa silage) and residual sugars are potential available substrates for the growth of aerobic microorganisms [41]. The large proportion of alfalfa silage in the AS60 group directly provided a stable acidic environment and reduced the consumption of WSCs during ensiling. Although the AS40 group did not have a lower lactic acid content or higher protective volatile fatty acid (acetic acid, propionic acid, and butyric acid) contents, the AS40 silages preserved less residual WSCs than the AS60 silages after fermentation. Higher remaining concentrations of unfermented sugars facilitate aerobic microbial growth [42], which may be the main reason for the lower aerobic stability of the AS60 group than the AS40 group. Furthermore, the AS40 silages showed significantly higher NH_3_-N contents than the AS60 silages after 5 days of aerobic exposure. Kung et al. [43] found that a higher ammonia content could significantly improve the aerobic stability of silage, which could also explain the higher aerobic stability of the AS40 group. Correspondingly, the molds were detected later in the AS40 group than in the AS60 group (3 days vs. 7 days). Therefore, it is optimal to develop a stable acidic environment during ensiling by adjusting the proportion of alfalfa silage in the TMR, which may help reduce potential substrates for aerobic microorganisms and enhance the aerobic stability of TMR silage.

## 5. Conclusions

A higher alfalfa silage content improved the digestibility of TMR silage in the present study. Unlike the acidic environment created directly by 60% alfalfa silage, when 40% alfalfa silage was added, the TMR developed a stable acidic environment during ensiling, and the aerobic stability of the TMR silage was also improved. Reducing the alfalfa silage content to 20% might increase the risk of clostridial spoilage and significantly reduce the quality of the TMR silage. Therefore, adding 40% alfalfa silage to TMR is optimal, which can achieve both good fermentation quality and considerable resistance to aerobic deterioration in TMR silage.

## Figures and Tables

**Table 1:** Chemical composition and fermentation characteristics of ingredients used for the total mixed ration.

Item ^1^	Alfalfa Silage	Corn Cob	Corn Grain	Mixed Concentrate ^2^
Dry matter (g·kg^−1^ FM)	473.63	940.04	887.88	921.03
Crude protein (g·kg^−1^ DM)	152.77	35.87	83.01	325.29
Neutral detergent fiber (g·kg^−1^ DM)	416.43	810.26	98.50	263.31
Acid detergent fiber (g·kg^−1^ DM)	306.29	417.88	38.09	110.84
pH	4.19	/	/	/
Lactic acid (g·kg^−1^ DM)	61.73	/	/	/
Acetic acid (g·kg^−1^ DM)	5.55	/	/	/
Propionic acid (g·kg^−1^ DM)	11.14	/	/	/
Butyric acid (g·kg^−1^ DM)	ND	/	/	/
Ammonia nitrogen (g·kg^−1^ TN)	15.01	/	/	/

ND, not detected. ^1^ FM, fresh matter; DM, dry matter; TN, total nitrogen. ^2^ The mixed concentrate was produced by Botai Company (Guyuan, China) and was composed of rapeseed meal, soybean meal, cotton meal, corn gluten meal, corn peel, distiller’s dried grains, and vitamin–mineral mix.

**Table 2:** Ingredient composition and chemical composition of the total mixed ration.

Item ^1^	Treatment ^3^			SEM	*p*-Value
	AS60	AS40	AS20		
Ingredient compositions (g·kg^−1^ DM)					
Alfalfa silage	600	400	200		
Corn cob	60	210	360		
Corn grain	240	290	340		
Mixed concentrate ^2^	100	100	100		
Total	1000	1000	1000		
Chemical compositions					
Dry matter (g·kg^−1^ FM)	569.12 ^A^	537.61 ^B^	501.36 ^C^	9.86	<0.001
Crude protein (g·kg^−1^ DM)	151.54 ^A^	132.54 ^B^	97.61 ^C^	8.00	<0.001
Water-soluble carbohydrate (g·kg^−1^ DM)	12.92	14.94	14.35	1.77	0.917
Neutral detergent fiber (g·kg^−1^ DM)	359.14 ^B^	386.90 ^AB^	418.88 ^A^	10.79	0.047
Acid detergent fiber (g·kg^−1^ DM)	239.49	232.95	219.01	4.88	0.234

Means within the same row with different superscript letters (A–C) differ significantly from each other (*p* < 0.05). SEM, standard error of the mean. ^1^ DM, dry matter; FM, fresh matter. ^2^ The mixed concentrate was produced by Botai Company (Guyuan, China) and was composed of rapeseed meal, soybean meal, cotton meal, corn gluten meal, corn peel, distiller’s dried grains, and vitamin–mineral mix. ^3^ AS60, TMR containing 60% alfalfa silage (DM); AS40, TMR containing 40% alfalfa silage (DM); AS20, TMR containing 20% alfalfa silage (DM).

**Table 3:** Changes in fermentative characteristics during ensiling of the TMR silages.

Item ^1^	Treatment ^2^	Days of Ensiling				SEM	*p*-Value ^3^		
		0	7	14	30		D	T	D × T
pH	AS60	4.44 ^b^	4.43 ^a^	4.46 ^b^	4.42 ^b^	0.01	<0.001	<0.001	<0.001
	AS40	4.50 ^bA^	4.30 ^bB^	4.28 ^cBC^	4.22 ^cC^				
	AS20	4.79 ^aA^	4.35 ^bC^	4.62 ^aB^	4.52 ^aB^				
LA	AS60	51.80 ^aAB^	45.29 ^B^	65.08 ^aA^	55.62 ^AB^	1.40	<0.001	<0.001	0.046
	AS40	39.83 ^bB^	24.22 ^C^	62.83 ^aA^	65.41 ^A^				
	AS20	18.47 ^cB^	23.31 ^B^	41.81 ^bA^	45.16 ^A^				
AA	AS60	2.95 ^B^	1.79 ^B^	2.36 ^bB^	11.13 ^A^	0.23	<0.001	0.050	0.021
	AS40	2.67 ^AB^	0.92 ^B^	3.24 ^aAB^	6.17 ^A^				
	AS20	2.28 ^B^	1.34 ^B^	3.08 ^aB^	6.86 ^A^				
PA	AS60	9.43 ^aAB^	7.81 ^aB^	11.92 ^aA^	12.03 ^aA^	0.25	<0.001	<0.001	0.216
	AS40	7.75 ^bA^	3.07 ^bB^	9.29 ^abA^	8.67 ^bA^				
	AS20	4.34 ^cB^	2.07 ^bB^	8.84 ^bA^	9.35 ^abA^				
BA	AS60	ND	ND	ND	ND	0.12	<0.001	<0.001	<0.001
	AS40	ND	ND	ND	ND				
	AS20	ND	ND	4.41 ^B^	10.3 ^A^				
NH_3_-N	AS60	10.85 ^aB^	2.91 ^C^	29.11 ^bA^	24.08 ^bA^	0.93	<0.001	<0.001	<0.001
	AS40	6.06 ^bB^	2.54 ^B^	33.60 ^bA^	26.95 ^bA^				
	AS20	4.84 ^bB^	3.18 ^B^	66.20 ^aA^	63.98 ^aA^				
V-score	AS60	92.02 ^bAB^	94.16 ^bA^	90.66 ^aB^	90.00 ^aB^	0.47	<0.001	<0.001	<0.001
	AS40	93.52 ^bB^	98.47 ^abA^	91.90 ^aB^	91.65 ^aB^				
	AS20	96.44 ^aA^	98.92 ^aA^	58.05 ^bB^	47.32 ^bC^				

Means within the same row (A–D) or within the same column (a–c) with different superscript letters differ significantly from each other (*p* < 0.05). SEM, standard error of the mean. ND, not detected. ^1^ LA, lactic acid (g·kg^−1^ DM); AA, acetic acid (g·kg^−1^ DM); PA, propionic acid (g·kg^−1^ DM); BA, butyric acid (g·kg^−1^ DM); NH_3_-N, ammonia nitrogen (g·kg^−1^ TN); V-score was used to evaluate the silage quality according to the volatile fatty acid and NH_3_-N contents, with the samples divided into three ranks: superior (81–100), good (60–80), and bad (<60); TN, total nitrogen; DM, dry matter. ^2^ AS60, TMR containing 60% alfalfa silage (DM); AS40, TMR containing 40% alfalfa silage (DM); AS20, TMR containing 20% alfalfa silage (DM). ^3^ D, effect of ensiling days; T, effect of treatment; D × T, interaction between ensiling days and treatment.

**Table 4:** Chemical compositions and in vitro digestibility of the TMR silages after 30 days of ensiling.

Item ^1^	Treatment ^2^			SEM	*p*-Value
	AS60	AS40	AS20		
Chemical compositions					
Dry matter (g·kg^−1^ FM)	555.25 ^A^	520.35 ^B^	459.81 ^C^	14.21	<0.001
Crude protein (g·kg^−1^ DM)	153.62 ^A^	135.83 ^B^	117.80 ^C^	5.21	<0.001
Water-soluble carbohydrate (g·kg^−1^ DM)	17.19 ^A^	7.06 ^B^	4.33 ^B^	2.02	<0.001
Neutral detergent fiber (g·kg^−1^ DM)	360.56 ^B^	371.05 ^B^	426.15 ^A^	11.42	0.009
Acid detergent fiber (g·kg^−1^ DM)	237.56	218.69	239.41	5.36	0.236
DM losses (g·kg^−1^ DM)	26.39 ^C^	35.58 ^B^	93.80 ^A^	6.17	<0.001
In vitro degradability					
DMD (g·kg^−1^)	583.73 ^A^	570.10 ^AB^	534.72 ^B^	8.94	0.037
NDFD (g·kg^−1^)	331.60	325.89	323.86	14.19	0.980
In vitro gas production parameters					
V_24h_ (mL)	58.60	56.10	51.86	1.90	0.394
V_48h_ (mL)	64.82	66.94	65.59	1.93	0.925
V(∞) (mL)	63.82	65.66	64.26	1.87	0.935
*k* (mL·h^−1^)	4.16	3.12	2.88	0.29	0.166

Means within the same row (A–C) with different superscript letters differ significantly from each other (*p* < 0.05). SEM, standard error of the mean. ^1^ FM, fresh matter; DM, dry matter; DMD, dry matter digestibility; NDFD, neutral detergent fiber digestibility; V_24h_, 24 h cumulative gas production; V_48h,_ 48 h cumulative gas production; V(∞), maximal cumulative gas production; *k*, maximum gas production rate. ^2^ AS60, TMR containing 60% alfalfa silage (DM); AS40, TMR containing 40% alfalfa silage (DM); AS20, TMR containing 20% alfalfa silage (DM).

**Table 5:** Changes in fermentative characteristics and chemical compositions of the TMR silages after exposure to air.

Item ^1^	Treatment ^2^	Days of Air Exposure					SEM	*p*-Value ^3^		
		0	1	3	5	7		D	T	D × T
Fermentative characteristics										
pH	AS60	4.42 ^bC^	4.43 ^bC^	4.47 ^C^	4.70 ^abB^	7.29 ^aA^	0.05	<0.001	<0.001	<0.001
	AS40	4.22 ^cC^	4.24 ^cC^	4.39 ^C^	5.45 ^aB^	6.86 ^aA^				
	AS20	4.52 ^a^	4.54 ^a^	4.5	4.54 ^b^	4.59 ^b^				
LA	AS60	55.62 ^A^	54.41 ^abA^	70.04 ^aA^	65.26 ^A^	31.97 ^bB^	1.72	0.025	0.001	0.027
	AS40	65.41 ^AB^	79.92 ^aA^	69.91 ^aAB^	60.22 ^AB^	47.43 ^abB^				
	AS20	45.16	42.23 ^b^	46.74 ^b^	46.44	52.66 ^a^				
AA	AS60	11.13	9.69	10.99 ^a^	7.61	7.91 ^a^	1.33	0.126	<0.001	0.044
	AS40	6.17 ^AB^	7.78 ^A^	3.14 ^cBC^	2.34 ^BC^	1.49 ^bC^				
	AS20	6.86	5.95	6.61 ^b^	7.55	8.37 ^a^				
PA	AS60	12.03 ^a^	10.78	13.18 ^a^	12.45	11.40	0.29	0.599	<0.001	0.624
	AS40	8.67 ^b^	9.43	8.09 ^b^	9.95	6.77				
	AS20	9.35 ^ab^	7.65	8.4 ^b^	9.04	9.27				
BA	AS60	ND	ND	ND	ND	ND	0.22	0.777	<0.001	0.885
	AS40	ND	ND	ND	ND	ND				
	AS20	10.3	7.96	8.73	10.08	9.61				
NH_3_-N	AS60	24.08 ^bB^	29.41 ^bB^	22.67 ^bB^	23.71 ^bB^	76.47 ^abA^	2.49	<0.001	0.001	<0.001
	AS40	26.95 ^bB^	36.81 ^bB^	26.28 ^bB^	48.13 ^aB^	138.56 ^aA^				
	AS20	63.98 ^aAB^	71.09 ^aA^	49.63 ^aB^	54.49 ^aAB^	61.58 ^bAB^				
V-score	AS60	90.00 ^aA^	90.00 ^aA^	90.00 ^bA^	90.00 ^aA^	85.54 ^aB^	0.78	0.001	<0.001	0.014
	AS40	91.65 ^aA^	90.00 ^aA^	92.90 ^aA^	91.29 ^aA^	68.83 ^abB^				
	AS20	47.32 ^b^	47.84 ^b^	50.27 ^c^	50.46 ^b^	47.68 ^b^				
Chemical compositions										
DM	AS60	555.26 ^aC^	565.42 ^aAB^	573.25 ^aAB^	581.11 ^aA^	566.84 ^aAB^	1.33	0.003	<0.001	0.035
	AS40	520.35 ^b^	519.13 ^b^	529.59 ^b^	526.51 ^b^	514.82 ^b^				
	AS20	459.81 ^cC^	469.16 ^cBC^	468.84 ^cBC^	479.78 ^cAB^	490.22 ^bA^				
CP	AS60	153.62 ^a^	151.99 ^a^	154.76 ^a^	153.11 ^a^	151.78 ^a^	0.52	0.247	<0.001	0.066
	AS40	135.83 ^bA^	138.59 ^bA^	139.67 ^bA^	138.57 ^bA^	129.43 ^bB^				
	AS20	117.79 ^c^	114.71 ^c^	117.94 ^c^	117.50 ^c^	120.14 ^c^				
WSC	AS60	17.19 ^aAB^	20.00 ^aA^	18.68 ^aAB^	16.31 ^aAB^	10.38 ^aB^	0.43	0.043	<0.001	0.204
	AS40	7.06 ^bAB^	9.53 ^bA^	6.35 ^bB^	6.47 ^abB^	7.24 ^abAB^				
	AS20	4.33 ^b^	4.88 ^c^	3.84 ^c^	3.66 ^b^	3.54 ^b^				

Means within the same row (A–C) or within the same column (a–c) with different superscript letters differ significantly from each other (*p* < 0.05). SEM, standard error of the mean. ^1^ LA, lactic acid (g·kg^−1^ DM); AA, acetic acid (g·kg^−1^ DM); PA, propionic acid (g·kg^−1^ DM); BA, butyric acid (g·kg^−1^ DM); NH_3_-N, ammonia nitrogen (g·kg^−1^ TN); V-score was used to evaluate the silage quality according to the volatile fatty acid and NH_3_-N contents; TN, total nitrogen; FM, fresh matter; DM, dry matter (g·kg^−1^ FM); CP, crude protein (g·kg^−1^ DM); WSC, water-soluble carbohydrate (g·kg^−1^ DM). ^2^ AS60, TMR containing 60% alfalfa silage (DM); AS40, TMR containing 40% alfalfa silage (DM); AS20, TMR containing 20% alfalfa silage (DM). ^3^ D, effect of aerobic exposure days; T, effect of treatment; D × T, interaction between aerobic exposure days and treatment.

**Table 6:** Changes in microbial compositions of the TMR silages after exposure to air.

Item ^1^	Treatment ^2^	Days of Air Exposure					SEM	*p*-Value ^3^		
		0	1	3	5	7		D	T	D × T
LAB (log_10_ cfu·g^−1^ FM)	AS60	7.26 ^bB^	6.88 ^bB^	6.83 ^cB^	6.96 ^bB^	7.87 ^bA^	0.04	<0.001	<0.001	<0.001
	AS40	6.87 ^cC^	6.78 ^bC^	7.11 ^bC^	8.02 ^aB^	9.87 ^aA^				
	AS20	7.62 ^aC^	8.01 ^aB^	7.69 ^aC^	8.27 ^aA^	8.4 ^bA^				
Yeast (log_10_ cfu·g^−1^ FM)	AS60	4.60 ^aB^	3.41 ^C^	5.48 ^aB^	6.68 ^bA^	7.32 ^aA^	0.07	<0.001	<0.001	<0.001
	AS40	3.38 ^bC^	3.21 ^C^	6.21 ^aB^	7.74 ^aA^	7.93 ^aA^				
	AS20	3.09 ^bBC^	2.22 ^C^	3.7 ^bAB^	4.2 ^cA^	4.1 ^bA^				
Mold (log_10_ cfu·g^−1^ FM)	AS60	<2.00 ^D^	<2.00 ^D^	2.90 ^aC^	4.06 ^aB^	4.58 ^aA^	0.03	<0.001	<0.001	<0.001
	AS40	<2.00 ^B^	<2.00 ^B^	<2.00 ^bB^	<2.00 ^bB^	4.96 ^aA^				
	AS20	<2.00	<2.00	<2.00 ^b^	<2.00 ^b^	<2.00 ^b^				
Coliform bacteria (log_10_ cfu·g^−1^ FM)	AS60	6.98 ^bA^	6.38 ^abB^	5.5 ^aC^	4.19 ^aD^	5.23 ^aC^	0.05	<0.001	0.008	<0.001
	AS40	6.56 ^bA^	5.99 ^bAB^	5.32 ^aB^	3.70 ^C^	5.79 ^aB^				
	AS20	7.61 ^aA^	6.77 ^aB^	4.62 ^bC^	3.22 ^cE^	3.9 ^bD^				

Means within the same row (A–E) or within the same column (a–c) with different superscript letters differ significantly from each other (*p* < 0.05). SEM, standard error of the mean. ^1^ LAB, lactic acid bacteria; cfu, colony-forming units; FM, fresh matter. ^2^ AS60, TMR containing 60% alfalfa silage (DM); AS40, TMR containing 40% alfalfa silage (DM); AS20, TMR containing 20% alfalfa silage (DM). ^3^ D, effect of aerobic exposure days; T, effect of treatment; D × T, interaction between aerobic exposure day and treatment.

## Data Availability

Data is contained within the article.
